# Medium-Term Function of a 3D Printed TCP/HA Structure as a New Osteoconductive Scaffold for Vertical Bone Augmentation: A Simulation by BMP-2 Activation

**DOI:** 10.3390/ma8052174

**Published:** 2015-04-28

**Authors:** Mira Moussa, Jean-Pierre Carrel, Susanne Scherrer, Maria Cattani-Lorente, Anselm Wiskott, Stéphane Durual

**Affiliations:** 1 Division of Fixed Prosthodontics and Biomaterials, University of Geneva, University Clinics of Dental Medicine, 19, rue Barthélemy-Menn, Geneva 1205, Switzerland; E-Mails: mira.moussa@unige.ch (M.M.); susanne.scherrer@unige.ch (S.S.); maria.cattani-lorente@unige.ch (M.C.-L.); anselm.wiskott@unige.ch (A.W.); 2 Department of Maxillofacial and Oral Surgery, Division of Oral and Maxillofacial Pathology (HUG), University Clinics of Dental Medicine, 19, rue Barthélemy-Menn, Geneva 1205, Switzerland; E-Mail: jean-pierre.carrel@unige.ch

**Keywords:** bone substitute block, 3D-printing, BMP-2, animal experiments, guided tissue regeneration, bone regeneration

## Abstract

**Introduction:** A 3D-printed construct made of orthogonally layered strands of tricalcium phosphate (TCP) and hydroxyapatite has recently become available. The material provides excellent osteoconductivity. We simulated a medium-term experiment in a sheep calvarial model by priming the blocks with BMP-2. Vertical bone growth/maturation and material resorption were evaluated. **Materials and methods:** Titanium hemispherical caps were filled with either bare- or BMP-2 primed constructs and placed onto the calvaria of adult sheep (*n* = 8). Histomorphometry was performed after 8 and 16 weeks. **Results:** After 8 weeks, relative to bare constructs, BMP-2 stimulation led to a two-fold increase in bone volume (Bare: 22% ± 2.1%; BMP-2 primed: 50% ± 3%) and a 3-fold decrease in substitute volume (Bare: 47% ± 5%; BMP-2 primed: 18% ± 2%). These rates were still observed at 16 weeks. The new bone grew and matured to a haversian-like structure while the substitute material resorbed via cell- and chemical-mediation. **Conclusion:** By priming the 3D construct with BMP-2, bone metabolism was physiologically accelerated, that is, enhancing vertical bone growth and maturation as well as material bioresorption. The scaffolding function of the block was maintained, leaving time for the bone to grow and mature to a haversian-like structure. In parallel, the material resorbed via cell-mediated and chemical processes. These promising results must be confirmed in clinical tests.

## 1. Introduction

The contemporary approach to regeneration is using resorbable matrices as scaffolds to guide the growth of new tissue. In the initial stages, the scaffold induces and guides cell growth and development. Later it supports the newly formed tissue while progressively resorbing. Ideally, the products of the resorptive process would be incorporated as natural components into the neosynthesized tissue.

Within the specific domain of bone reconstruction, the optimal matrix is “osteopromotive”, that is, it functions as a pattern for osteoconduction, releases growth factors and provides scaffolding to maintain the volume in which osteogenesis will take place [1]. At the cellular level, the scaffold is perceived as a form of extracellular matrix which promotes both cell migration and stabilization in a controlled and physiologically effective manner. To this effect, the scaffold should present a moderate (2–3 µm) surface roughness to permit cell adhesion and guidance [2,3]. At the macroscale level (0.1–0.4 mm range), the scaffold must be porous (about 60 vol%) so that it can be penetrated by cells and new vessels. Further, the pores must be interconnected for the osteoconductive process to spread into the depth of the scaffold [4,5]. Finally, the scaffold should resorb at a rate that harmonizes with the formation and maturation of new bone. In its superlative form, the scaffold would also support physiological loads in the early stages of the process. These loads would then be transferred to the newly formed bone as it matures while the scaffold disappears.

Calcium orthophosphates bioceramics, e.g. hydroxyapatite (HA), α- or β-tricalciumphosphates (α- or β-TCP) are chemically and structurally similar to the natural components of bone and teeth [6]—a resemblance which provides them with excellent osteoconductive properties [7]. As their rate of resorption can be adjusted by adjusting the Ca/P and HA/TCP ratios [8], calcium phosphates enter into the composition of many synthetic bone substitutes. A most prominent representative in this category is biphasic calcium phosphate (BCP)—a combination of HA and TCP formulated as an optimum balance between the most stable form of HA and most soluble form of TCP [9]. Recently, a 3D-printed construct (OsteoFlux^®^) made of BCP has been developed to form a regular arrangement of orthogonally layered cylindrical filaments with inner channels of 250 µm. In a sheep calvarial model, the osteoconductivity of this construct was 2–4 times greater 8 weeks after implantation than particulate bone substitutes of natural- (bovine bone) or synthetic (β-TCP) origin [10]. At 16 weeks, bone formation had largely evened out for all three materials. Still, four month was a duration that was sufficient to evaluate the potential for osteoconductivity, but insufficient for the bone to fully mature and the material to resorb.

Bone morphogenetic protein 2 (BMP-2) is a potent activator of bone metabolism [11]. Therefore, our working hypothesis was that adding BMP-2 to the constructs would accelerate bone metabolism and thus permit an investigation of bone maturation and remodeling as well as material resorption within the 16 weeks of the study. Our objective, therefore, was to histologically and histomorphometrically evaluate bone maturation on OsteoFlux^®^ constructs and their resorption after an initial stimulation with BMP-2 in a model of guided bone regeneration in sheep.

## 2. Materials and Methods

### 2.1. Experimental Design

This experiment was a subproject of a larger study conducted on 12 sheep in which the osteoconductive performance of 3D printed constructs was compared to two other bone substitutes using titanium caps placed on sheep skulls [12]. Six locations were available (occipital left and right, median left and right, frontal left and right). Six randomly allotted titanium hemispheres filled with (i) 3D printed constructs; (ii) 3D printed constructs + BMP-2; (iii) particulate bone substitute of bovine origin; (iv) particulate bone substitute of synthetic origin or (v) plain blood coagulum (negative control) were placed on the calvarium of each animal. Randomization was obtained by drawing lots with the following constraints. (i) Each sheep was to receive at least one of the three substitutes but (ii) no sheep could be fitted with more than two samples of the same substitute; (iii) each of the three substitutes was to be placed at least once in one of the six positions; (iv) the four controls (no substitute) had to be placed on different animals and (v) distributed over the occipital, the median and the frontal sites. Six animals were euthanized after 8 weeks and six after 16 weeks. The skulls were block-sectioned and subjected to histomorphometric analysis. Outcomes were analyzed in terms of the relative volumes of bone substitute and new bone tissue.

The data on the synthetic construct and the particulate bone substitutes have been published elsewhere [10]. The following report specifically addresses the response of the synthetic construct with and without BMP-2.

#### **Samples** 

The following conditions were evaluated (Table 1):

1. **3D-printed constructs** (OsteoFlux^®^, Vivos-Dental, CH), **no BMP added**, serving as baseline, *n* = 8. These constructs are made of orthogonally layered cylindrical filaments, 350 µm in diameter separated by spaces 250 µm in width. Upon printing, the filaments are deposited as a high viscosity paste which progressively sets to a hard consistency. The paste is synthetic calcium phosphate with a calcium-to-phosphate ratio of 1.43 (range: 1.35 to 1.5). Calcium phosphates are present in the form of α-TCP and microcrystalline, calcium deficient HA. The constructs’ macroporosity ranges between 40% and 50% and the total porosity between 50% and 65% [10].

2. **3D-printed block, BMP-2 added**, *n* = 8. Recombinant human BMP-2 (Inductos^®^, Medtronic, USA) was prepared under sterile conditions according to the manufacturer’s specifications (1.5 mg/mL). At surgery it was spread as a single dose of 100 µg onto the 3D-printed block. After complete absorption, autologous blood was added prior to placement of the titanium hemisphere onto the skull.

For the sake of simplicity, plain OsteoFlux^®^ specimens and OsteoFlux^®^ + BMP-2 specimens will be referred to as OF-plain and OF-BMP-2 hereafter.

**Table 1:** Specimens analyzed.

Healing period	Samples	Number of specimen analyzed (n)
8 weeks	Coagulated blood	4
	3D-printed block (OF-plain)	8
	3D-printed block + BMP-2 (OF-BMP-2)	8
16 weeks	Coagulated blood	4
	3D-printed block (OF-plain)	8
	3D-printed block + BMP-2 (OF-BMP-2)	8
Total	Total placed: 40	Total analyzed: 40

### 2.2. Hemispheres and Placement of Bone Substitutes

**Figure 1:**
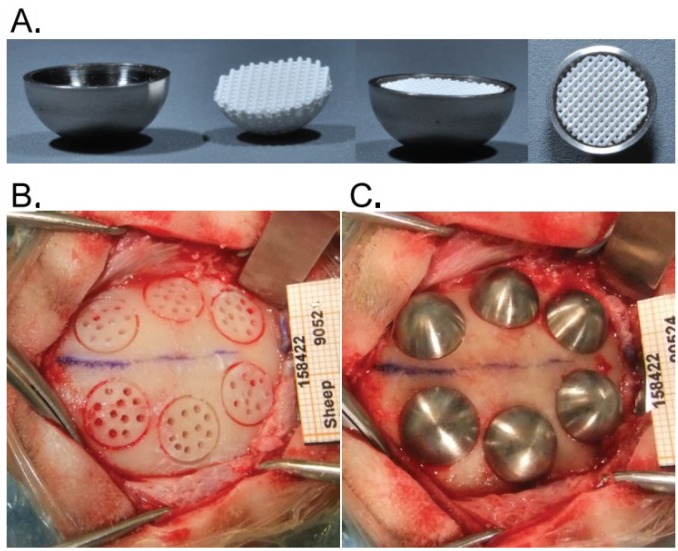
(**A**) Titanium hemispheres filled with 3D printed construct; (**B**) circular grooves with transcortical perforations prior to the fixation of the hemispheres filled with bone substitute (**C**).

The hemispheres were machined out of titanium (cpTi-gr2) with an inner diameter of 10 mm, an outer diameter of 11 mm and a height of 5 mm. OF constructs were printed to a perfect fit inside the hemispheres (Figure 1A) and were gamma sterilized before use.

At the time of surgery, the OF constructs were placed into the hemispheres and impregnated with autologous blood. BMP-2 was added to the test specimens. The negative control spheres were merely filled with blood (no construct). The hemispheres were placed onto the skull immediately after being filled.

### 2.3. Animals

Twelve adult BMC sheep (female, 2.5–4 years, 69–87 kg) were included in the study (Eymin Breeding, Seyssuel, France). The animals underwent an acclimation period of one week prior to surgery. The experiment was conducted in a dedicated facility (NAMSA, Chasse sur Rhône, F (ISO/CEI 17025) [13]). In line with European requirements (European Directive 2010/63/EU [14]), all animal experiments were approved by veterinary committees (NAMSA Ethical Committee and French Ministry of Agriculture).

### 2.4. Surgical Procedure

Placement of the titanium caps was performed under general anesthesia. The animals were deprived of food (24 h) and water (12 h) prior to surgery to prevent vomiting. A prophylactic antibiotic coverage was dispensed 1 day before and up to 2 weeks after the surgery (amoxicillin-Duphamox, Pfizer, Fort Dodge, IA, USA, i.m. every 2 days (15 mg/kg) and enrofloxacin-Baytril 5%, Bayer Pharma, Leverkusen, Germany, s.c. daily (5 mg/kg)). The animals first received an analgesic treatment (flunixine-Meflosyl i.m. Pfizer/Fort Dodge, NY, USA (2 mg/kg), and buprenorphine-Buprecare s.c. (0.01 mg/kg), Animalcare, York, England). Anesthesia was induced by an intravenous injection of a mixture of thiopental-Nesdonal (750 mg, Merial, Lyon, France)—Pentobarbital sodium (273 mg, CEVA, Libourne, France)—Atropine sulfate (1 mg, Aguettant, Lyon, France) before intubation. Deep anesthesia was obtained using 2% isoflurane-Aerrane (Baxter, Deerfield, IL, USA) in pure oxygen. A rectal temperature probe and a rumen tube were placed. Heart function, temperature and oxygen saturation were monitored. Iso-volumetric conditions were maintained by infusion of a ringer lactate solution during the entire procedure.

The surgical areas were shaved and the skin was scrubbed with povidone iodine (Vetoquinol, Magny-Vernois, France), wiped with isopropyl alcohol, painted with povidone iodine and draped. First a midline incision was made through the skin, from the orbits to the external occipital protuberance. The temporalis muscles were elevated subperiosteally from the frontal and parietal bone and bilaterally retracted. On each side of the median suture, 3 circumferential grooves, 10 mm in diameter and approximately 0.5 mm in depth, were trephined under saline irrigation. In each of the resulting circles 11 holes that penetrated the medulla (1 mm in diameter, *ca.* 2 mm in depth) were drilled with a round bur to allow bone cell migration from the marrow to the surface. Thus in each animal, 6 grafting sites made of a cortical bone plate penetrated by 11 transcortical perforations were produced (Figure 1B). Primary stability of the hemispheres was ensured by the clipping effect of the titanium caps onto the bone (Figure 1C). Each animal received at least one of each bone substitute. Wound closure was carried out in 3 planes. The deeper tissues were closed using a discontinuous resorbable suture (Vicryl 3-0, Ethicon, Somerville, NJ, USA). A povidone iodine solution was applied before closing the subcutaneous tissues with the resorbable suture material. The skin was closed with a continuous non-resorbable suture (Prolene 3-0, Ethicon, Somerville, NJ, USA) and disinfected with a spray of oxytetracyclin (Oxytetrin, Summit, NJ, USA).

Post-operative pain was minimized by subcutaneous injections of buprenorphine (Buprecare, Animalcare, UK) 0.01 mg/kg, twice daily for 2 days. Inflammation was controlled for 7 days by daily intramuscular injections of flunixine (Meflosyl, Zoetis, Florham Park, NJ, USA) 2 mg/kg. The sutures were removed after complete healing of the skin. The wounds were disinfected until 2 days thereafter.

After sacrifice, the skulls were dissected and immersed in neutral buffer and 10% formalin.

### 2.5. Histological Preparation, Histopathologic and Histomorphometric Analysis

After complete fixation, the skulls were block-sectioned to isolate each hemisphere. The blocks were (i) rinsed for 3 h with tap water, (ii) dehydrated in alcohol solutions of increasing concentration; (iii) cleared in xylene and (iv) embedded in polymethyl methacrylate resin (Merck, Kenilworth, NJ, USA). Using a precision band saw (EXAKT system, Norderstedt, Germany), undecalcified specimens were obtained by sectioning the titanium caps perpendicular to the bone surface mid-way through the hemispheres. Then the specimens were thinned out to a thickness of 30–40 µm by microgrinding and polished (Mecapol P320, Presi, F, Grenoble, France). Finally they were stained with modified paragon. The sections were digitized with a light microscope (Eclipse 80i, Nikon, Tokyo, Japan) coupled to a digitizing camera (Allied Vision Technologies, D, Stadtroda, Germany). Single pictures of high power fields at 10x magnification were assembled to generate large overview images.

The outlines of bone substitute remnants and new bone tissue were scribed using a software for image analysis (Calopix viewer, Tribun, Châtillon, France). The structures of interest were delimited manually using a graphical pad on the entire surface inside the titanium caps, that is, from the external cortical limit of the bony bed to the internal limit of the caps. The amounts of new bone and bone substitute were expressed as percentages of the total volume under the hemispheres. Artifacts and/or broken tissues were excluded from the computations. The histopathologic evaluation was performed using the scoring system described in Table 2 (ISO 10993-6 standard [15]).

### 2.6. Statistical Analysis

The histomorphometrical data were checked for normal distribution and equivalence of variances. Unpaired t-tests were used to compare OF-plain (*n* = 8) to OF-BMP-2 (*n* = 8), at 8 and 16 weeks. For the analyses on horizontal segmentation, only groups within a slice were compared. The null hypothesis was rejected at *p* < 0.05.

## 3. Results

At necroscopy, macroscopically none of the 40 sites presented signs of inflammation. All hemispheres remained as placed both at 8 and 16 weeks.

### 3.1. Histopathologic Evaluation

Prior to histomorphometric analysis, a histopathologic quantification of inflammatory and bone cells was conducted. The scoring system and the resulting data are presented in Table 2 and Table 3. No inflammatory cell infiltrate was detected at any site. Regarding phagocytic cells however, that is those in charge of degradation and remodeling processes, the application of BMP-2 led to heavy infiltrates of macrophages after 8 weeks while only a few cells were observed in control specimens. After 16 weeks, the situation had evened out with a slight presence of macrophages both in experimental and control specimens. BMP-2 had no effect on osteoclasts and giant cells, which were rarely seen both at 8 or 16 weeks. Conversely there was a marked presence of osteoblasts on both surface types at 8 weeks, which slightly decreased after 16 weeks.

**Table 2:** Histopathologic scoring system (semi-quantitative).

Cell type/response	Score				
	0	1	2	3	4
Polymorphonuclear cells (PMN)	0	Rare, 1–5/phf	5–10/phf	Heavy infiltrate	Dense
Lymphocytes (Lc)	0	Rare, 1–5/phf	5–10/phf	Heavy infiltrate	Dense
Plasma cells (Plc)	0	Rare, 1–5/phf	5–10/phf	Heavy infiltrate	Dense
Macrophages (Ma)	0	Rare, 1–5/phf	5–10/phf	Heavy infiltrate	Dense
Giant cells/osteoclastic cells (Gc/Oc)	0	Rare, 1–2/phf	3–5/phf	Heavy infiltrate	Sheets
Osteoblasts (Ob)	0	Slight, equivalent to normal bone	Moderate, >normal bone	Marked, >normal bone	Highly marked
phf = per high powered (400×) field					

**Table 3:** Semi quantitative histopathologic evaluation (Scoring Table 2, *n* = 8, mean ± SD).

Time	Samples *n* = 8	Polymorphomuclear cells	Lymphocytes	Plasma cells	Macrophages	Giant Cells/ osteoclastic cells	Osteoblastic cells
8 weeks	OF-plain	0	0	0	1.1 ± 0.3	0.9 ± 0.3	2.6 ± 0.5
	OF-BMP-2	0	0	0	2.6 ± 0.5	1	3
16 weeks	OF-plain	0	0	0	1.3 ± 0.4	1	2 ± 0.5
	OF-BMP-2	0	0	0	1.6 ± 0.5	1.1 ± 0.3	1.8 ± 0.7

### 3.2. Histologic Evaluation

**Figure 2:**
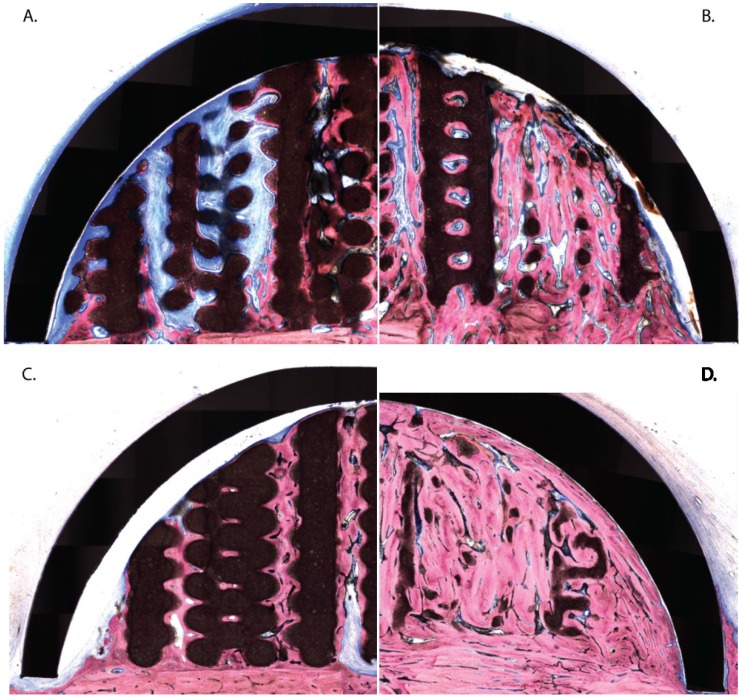
Hemi-slide showing the bone substitutes’ engraftment under the titanium caps at 8 and 16 weeks. (**A**) 3D construct (8 weeks); the construct appears as brown circles or rounded polygons. The newly formed bone (pink) sprouts from the transcortical perforations and the bony bed. It then develops into vertical columns up to the titanium caps. The remainder of the volume is filled with fibroid and osteoid tissue (blue); (**B**) BMP-2 primed construct (8 weeks); large portions are filled with new bone already entering the remodeling process. Some particles of bone substitute appear as largely degraded and intimately nested into the new bone. Other portions of bone substitute remain undegraded; (**C**) 3D construct (16 weeks); fibroid and osteoid tissue is replaced by maturing new bone. The bone substitute did not resorb; (**D**) BMP-2 primed construct (16 weeks); traces of bone substitute (largely imbricated into a mature bone) are still observable.

Generally, from 8 weeks on, the hemispheres were covered by a fibrous layer. The circular grooves that stabilized the hemispheres were often filled with new bone (Figure 2C,D) which somewhat lifted the caps out of their bone bed. As a consequence, on the inside of the caps a layer of connective tissue developed between the metal and the substitute materials.

(Figure 2A–C). In the negative control sites (plain blood coagulum) the hemispheres were almost empty although some minor bone growth was noted [10].

At 8 weeks, OF-plain samples showed evident signs of vertical and horizontal osteoconduction along the canals of the construct (Figure 2A). The new bone primarily grew out of the transcortical holes but also developed from the cortical bone bed and often spread to the top of the structure. The osteoid and the fibrous connective tissue were largely vascularized and showed marked osteoblastic activity. Large parts of the bone substitute were well integrated into the new bone tissue while others were lined by a thin layer of osteoid tissue and rows of osteoblasts. Signs of material degradation were observed at the base of the scaffold.

**Figure 3:**
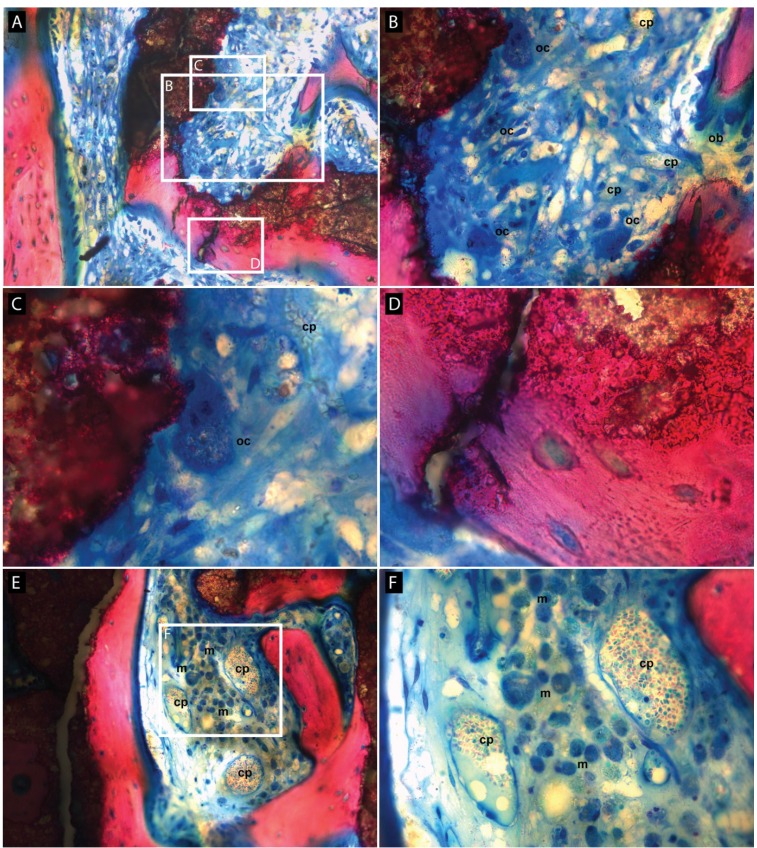
Higher magnifications of active zones of resorption in a BMP-2 primed construct (**A**,**E**) showing various processes of resorption of the scaffold at 8 weeks. The fibroid and osteoid tissue (blue) is highly vascularized (**B**,**C**,**F**), as shown by the broad presence of capillaries (cp), and is rich in plump macrophages (m) harboring intracytoplasmic debris; some osteoclasts (oc) are present and active (**B**,**C**). The bone substitute is not only degraded via cellular activity, but is also biochemically dissolved into the newly synthesized bone (**D**).

Adding BMP-2 led to the growth of large amounts of new bone that filled a vast surface. At 8 weeks, signs of maturity and remodeling were observable (Figure 2B). The remaining space was colonized by an active osteoid tissue. It also contained remnants of bone substitute which demonstrated evident and substantial signs of degradation, primarily mediated by macrophages (Figure 3A,E,F) and occasionally by osteoclasts (Figure 3A–C). A form of dissolution of the material into the newly formed bone tissue but without apparent cell mediation was also often observed (Figure 3A,D).

**Figure 4:**
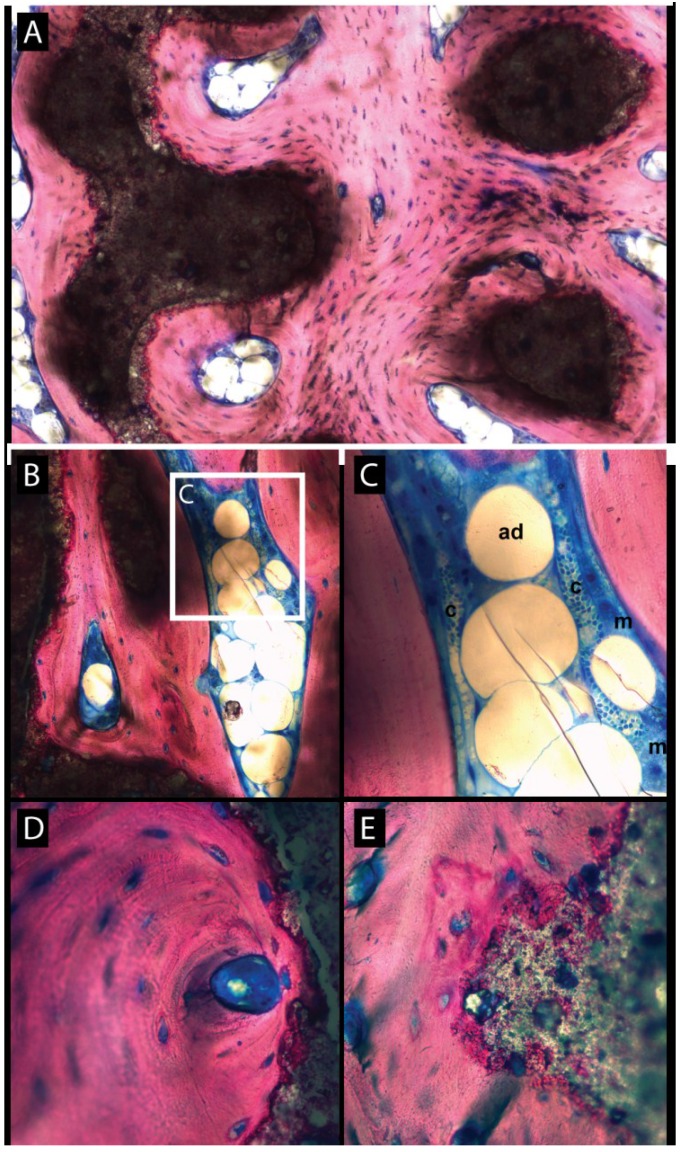
Higher magnifications showing mature bone at 16 weeks in BMP-2 primed constructs. (**A**) Presence of “haversian like” structures with adipocytic inclusions. Note the tight integration of the bone substitute remnants; (**B**) adipocytic inclusion within mature bone and higher magnification (**C**) showing adipocytes (ad), numerous capillaries (cp) and plump macrophages (m); (**D**) presence of newly formed osteons with central vasculature in close contact to the bone substitute that “dissolves” into the mature bone (**E**) in many locations.

At 16 weeks, the new bone had matured and had replaced the osteoid and connective tissue in the OF-plain samples (Figure 2C). The bone substitute was tightly surrounded by the new bone tissue but degradation was light. In OF-BMP-2 samples, the cap was entirely filled by new mature bone (Figure 2D). The remodeling and maturation process led to a homogenous tissue which merged with the native cortical bone. Haversian-like structures were occasionally observed (Figure 4A) and newly formed osteons developed close to substitute remnants (Figure 4D). A differentiation into fatty bone marrow was also noted in some lacunae (Figure 4A–C). Traces of bone substitute remained which progressively dissolved into the new bone tissue (Figure 4E.)

### 3.3. Histomorphometric Evaluation

**Figure 5:**
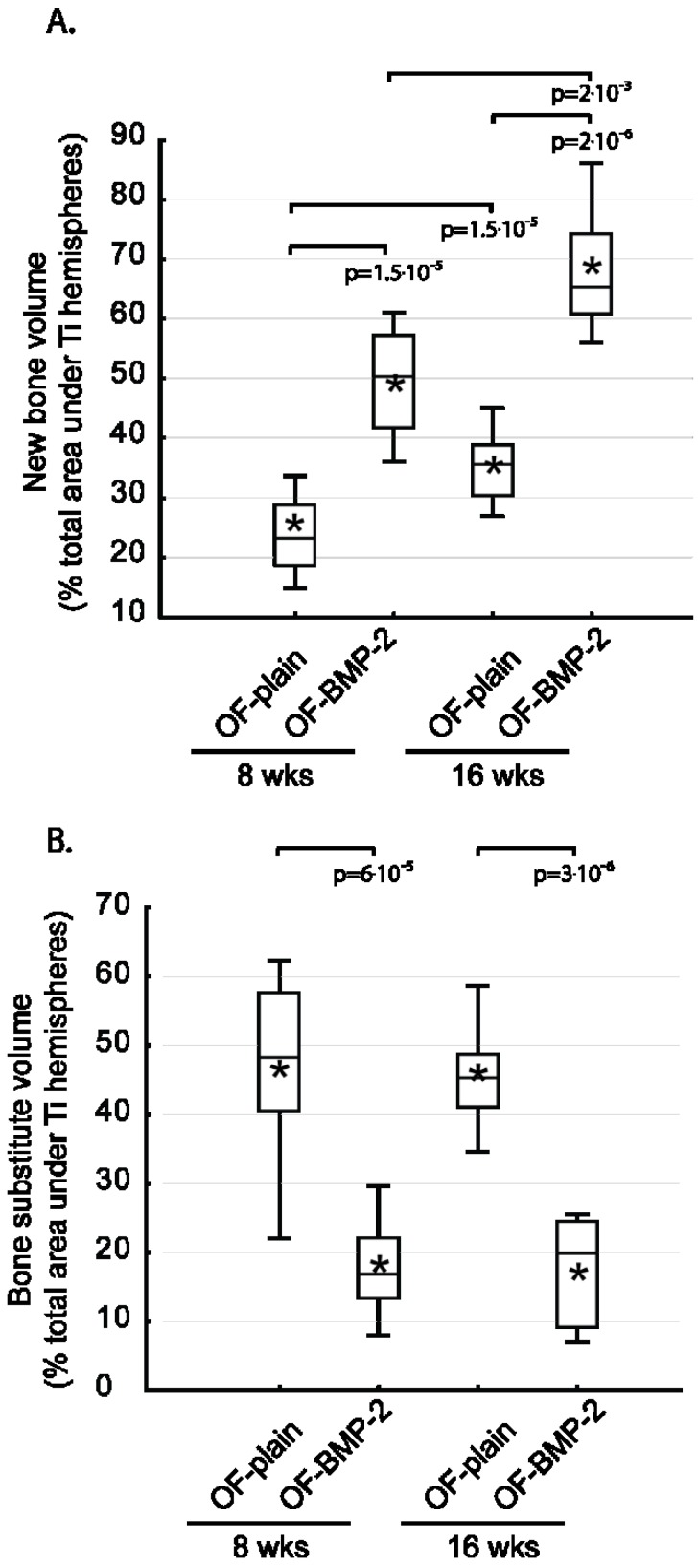
Histomorphometrical assessment of new bone volume (**A**) and volume of bone substitute (**B**) under the hemispheres at 8 and 16 weeks (*n* = 8 for each column). Brackets and p values indicate significant differences. Horizontal line: median. Boxes: 25%–75%. *: mean. Bars: range of non-outliers. (OF-plain: bare construct; OF-BMP-2: BMP-2 primed construct).

**Figure 6:**
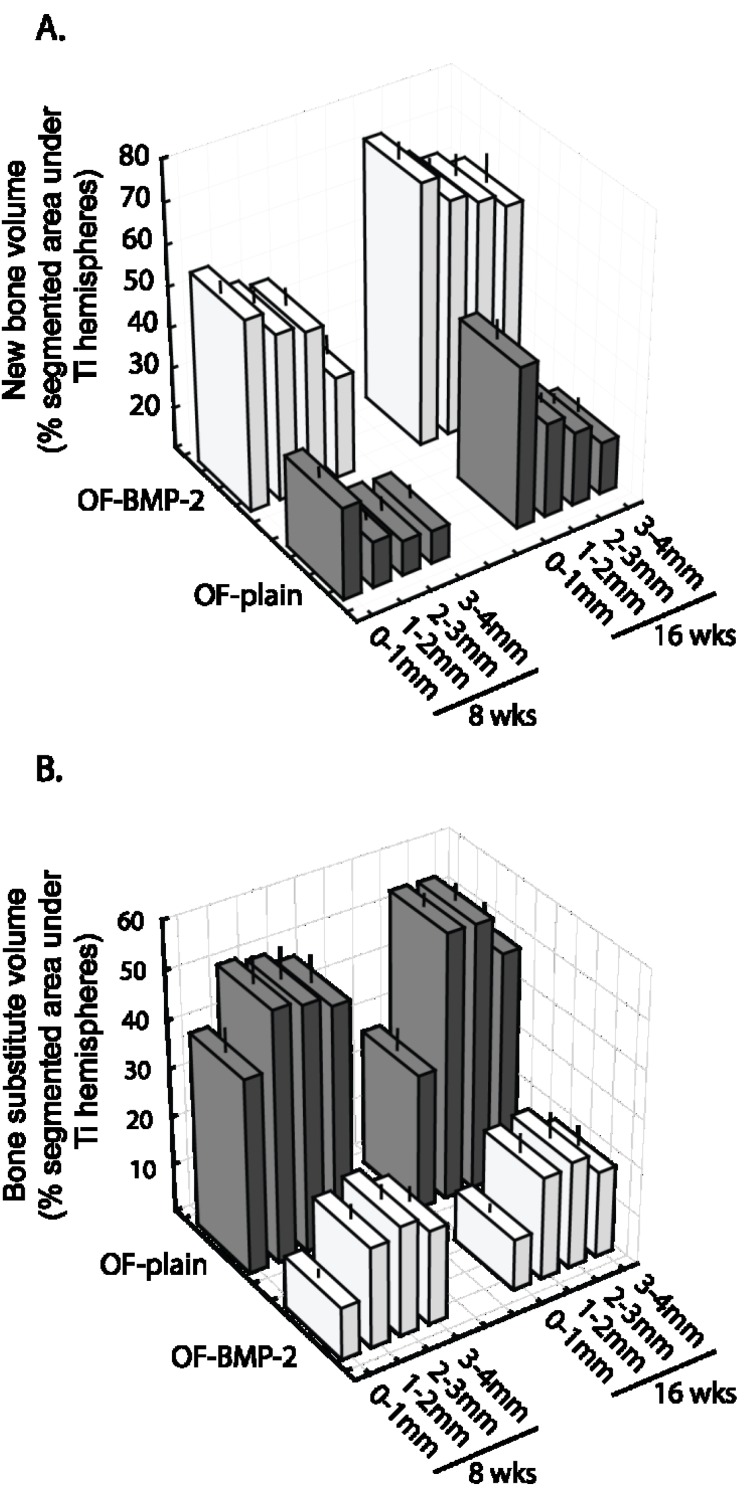
New bone volume (**A**) and bone substitute volume (**B**) under the hemispheres, segmented in 4 horizontal planes of 1 mm in height at 8 weeks and 16 weeks in OF-plain (grey) and OF-BMP-2 (white) samples. (*n* = 8 for each column). (Bars = SEM, OF-plain: bare construct; OF-BMP-2: BMP-2 primed construct).

The histomorphometric analyses addressed the bone substitute and the new bone tissue that had formed under the hemispheres. At 8 weeks, the new bone volume (NBV) in OF-plain specimens was 22% ± 2.1%. The addition of BMP-2 significantly stimulated bone growth by 2.2, NBV reaching 50% ± 3% (*p* = 1.5 × 10^−5^) (Figure 5A). This augmentation was observed from the base to the top of the scaffold (Figure 6A). At 16 weeks, a rate of growth of 1.5 was observed in both conditions, whatever the compartment analyzed (Figure 6A), NBV amounting to 35% ± 2% on OF-plain and 68% ± 4% on OF-BMP-2 samples (*p* = 2 × 10^−6^) (Figure 5A).

Regarding the volume occupied by the bone substitute (BSV), an opposite effect was observed. After 8 weeks, the addition of BMP-2 led to a material degradation that was 2.7 higher than that of OF-plain specimens (OF-plain: 47% ± 5%, OF-BMP-2: 18% ± 2%, *p* = 6 × 10^−5^) (Figure 5B). These ratios remained nearly constant after 16 weeks (OF-plain: 45% ± 2%; OF-BMP-2: 17% ± 3%; *p* = 3 × 10^−6^), the distribution of calculated values tending to be more constricted when compared to 8 weeks (Figure 5B). These differences were significant from 0 to 4 mm up to the bony bed, at 8 and 16 weeks (Figure 6B).

## 4. Discussion

The present work aimed at simulating the performance of a 3D-printed construct of bone substitute (OsteoFlux^®^) over a period of time beyond that observed under standard conditions. Bone metabolism was considerably accelerated by an initial stimulation with BMP-2. Osteoconduction, bone maturation as well as material resorption were histologically and histomorphometrically evaluated 8 and 16 weeks after implantation. Unstimulated constructs served as controls.

The calvarial model of a one-wall defect in which bone substitutes are maintained and protected by titanium caps was first developed for rabbits [16,17] and pigs [12]. The model has recently been transferred to sheep and proved satisfactory when comparing several bone substitutes [10] (sheep have been widely used in cranioplasty, distraction and reconstruction studies [18,19,20]).

The main advantages of this model are as follows: (i) several conditions may be compared simultaneously (e.g., different bone substitutes, addition of cytokines); (ii) in comparison to bone defect models, the cortical bone bed is preserved, thereby testing the true potential of a material for promoting bone augmentation; (iii) medullar cells may migrate towards the experimental via transcortical perforations.

The study herein described was a part of a larger study aimed at comparing a synthetic 3D construct to two other bone substitutes [10]. When investigating the osteoconductive potential of a material, a timeframe of 16 weeks is generally considered adequate [12,16,17]. Problematically, the response of phosphoceramics regarding their ability to resorb must be approached in timeframes from months to years [21]. Yet the current trend in animal testing is to minimize the number of animals and the duration of the experiments. For this reason, it was felt that that accelerating bone metabolism (*i.e*., bone synthesis and bone remodeling) was an elegant solution to investigate the process of bone maturation and material resorption at later stages of development.

The bone morphogenetic protein (BMP) family of cytokines and growth factors is known for their potential for osteoinduction, both orthotopically and ectopically. Among these, BMP-2 is a most prominent representative to the extent that it found clinical applications in bone repair [11]. Still, side effects such as heterotopic bone formation [22], bone resorption and inflammation [23], and cervical or gingival swelling [24] were noted. Due to the well-delineated zone of effect of BMP-2 in our setup, none of these signs were observed in our study (except for local bone overgrowth out of the caps).

BMP-2 is typically used at supraphysiological concentrations (in the tenths of a milligram range) to compensate for its proteolytic degradation. Therefore scaffolds that would release BMP-2 at a steady rate (instead of a one-time massive discharge) would be of great interest to (i) decrease dosages; (ii) maintain a sustained stimulatory effect on bone induction and (iii) decrease side effects [11]. In numerous clinical applications, collagen sponges were used. Still, due to their rapid degradation, they tend to release BMP-2 at high doses and over too short a time span. In contrast, incorporating BMP-2 into a phosphocalcic bioceramic may have dual advantages—first, a release of BMP-2 according to a natural flow, and second, a reinforcement of the device’s mechanical resistance [25,26]. In the present experiment, loading the 3D construct with 100 µg BMP-2 led to a bone growth that was twice that of controls while still retaining its normal characteristics in terms of physiological processes, that is, abundant neovascularization, important osteoblastic activity and matrix mineralization.

After sufficient amounts of new mineralized tissue have been formed, the next step consists in remodeling to set up the bone’s definitive architecture: structurally (haversian lamellae), morphologically (trabecular, cortical) and with regard to the overall anatomy. Eight weeks after implantation, the first signs of bone remodeling were observed in BMP-2 treated specimens. These became evident at 16 weeks, as shown by the presence of newly formed osteons, harversian like structures, adipocytic bone marrow inclusions and the merging of the new bone with the cortical bony bed.

In conclusion, the addition of 100 µg BMP-2 onto the scaffold succeeded in physiologically accelerating bone development, leading to the development of pre-mature haversian bone.

The ultimate goal of the study was to evaluate the 3D constructs regarding scaffolding for new bone as well as their ability to progressively resorb. The biodegradation process of phosphoceramics is a combination of chemical dissolution and cell-mediated resorption [6,27]. The chemical dissolution primarily depends on the nature and crystallinity of the ceramic [6,28]. On the biological end, the cells involved in the degradation process are mainly of the phagocytic lineage, that is, osteoclasts, giant cells, macrophages and monocytes [27].

After 16 weeks, the resorption of the material was 2–3 times higher in BMP-2 treated specimens but then nearly halted for the next two months. Hence, most material resorption took place during the first 8 weeks.

BMP-2 is known for its bifunctional stimulation, that is, it affects bone formation as well as bone resorption. The latter is mediated by osteoclasts which are activated via (i) the RANK-L/RANK/OPG regulation loop [29,30] or (ii) a direct interaction with BMP-2 receptors on osteoclasts [31,32]. High doses of BMP-2 activate both osteoblasts and osteoclasts at early healing stages (1 week to 1 month) (nonetheless there is a preponderance of osteoclasts in the very initial times [33]). Subsequently, resorption units, made of a pool of osteoclasts which are continuously renewed, are recruited and maintained for a period of several weeks to months [34].

In our study osteoclasts were occasionally observed in BMP-2 treated specimens but not in substantial amounts, neither were resorption units. Furthermore, the cortical bone bed, which normally would have been heavily remodeled by massive osteoclast infiltrations, remained largely intact. Therefore, it appears that no sizeable osteoclast-mediated bone/material resorption process was active in the first 8 weeks. In contrast, 2–3 times more macrophages harboring debris of material were found in close vicinity of BMP-2 treated scaffolds. Macrophages are known precursors of osteoclasts but may also degrade foreign materials on their own [27]. They are able to synthetize BMP-2 and thus activate bone synthesis [35] but their own response to BMP-2 is poorly documented. As observed here, the sequence of events takes place as if BMP-2 induced the recruitment and activation of macrophages to degrade the 3D construct instead of engaging the cells to differentiate into osteoclasts. Still, the process left sufficient time for the scaffold to support the migration of bone cells and the formation of new bone as demonstrated by the bone's morphology at 8 weeks (made of vertical columns reflecting the original scaffold structure, by now largely dissolved).

As mentioned above, phosphoceramics, and especially TCP, are known to dissolve in water [6,28]. Dissolution was observed both on controls and on BMP-2 specimens, but was not actually increased by BMP-2 treatment. During the last two months of the experiment, the material continued its resorption process, but at a very slow pace.

Finally, considering the ability of the 3D construct to support the formation of a mature bone, there was clear evidence that the construct had promoted a large amount of bone ingrowth during the first 8 weeks, followed by a remodeling and maturation process finally leading to a harversian-like bone. The construct’s remnants after 16 weeks were fully osteointegrated and showed signs of dissolution. Eventually the process should lead to the complete disappearance of the material. This, however, must be confirmed by further experiments. The anatomical shape of the newly synthesized bone was very regular, patterned by the scaffold and delimited by the titanium cap.

## 5. Conclusions

Priming a 3D printed construct with BMP-2 accelerated bone metabolism whereby this acceleration proceeded within physiological boundaries. The rate of vertical bone growth, bone maturation and material bioresorption nearly doubled after implantation in sheep calvaria. The scaffolding function of the constructs was maintained, thus leaving sufficient time for the bone to grow and mature until finally acquiring a haversian-like structure. In parallel, the biomaterial resorbed via cell mediated and chemical processes. These are promising results which must be confirmed in clinical tests.
